# FSH versus AMH: age-related relevance to ICSI results

**DOI:** 10.1186/s43043-021-00071-6

**Published:** 2021-08-17

**Authors:** Sameh Salama, Marwa Sharaf, Sondos M. Salem, Mazen Abdel Rasheed, Ehab Salama, Tamer Elnahas, Rehab Lotfy

**Affiliations:** 1 Reproductive Health and Family Planning Department, Medical Research Division, National Research Centre, 33 Al Bohouth Street, Dokki, Giza, Egypt; 2 Obstetrics and Gynaecology Department, Kasr El Eini Teaching Hospital, Cairo University, Cairo, Egypt

**Keywords:** Follicle stimulating hormone (FSH), Anti-Müllerian hormone (AMH), Intra-cytoplasmic sperm injection (ICSI)

## Abstract

**Background:**

Women’s fecundity is known to decrease with the increase in chronologic age. Several biomarkers of the ovarian reserve, including follicle stimulating hormone (FSH), anti Müllerian hormone (AMH), have been proposed as possible predictors for the response to controlled ovarian stimulation (COS). Although there are assumptions indicating that the relationship between age and ovarian reserve is highly variable and the potential different validity of ovarian reserve markers in women in different age groups remains to be demonstrated. The purpose of our study was evaluating FSH and AMH as potential predictors of response to controlled ovarian stimulation and prediction of intracytoplasmic sperm injection (ICSI) outcome according to age. This prospective study has been carried out on 218 women having ICSI cycles. Cases were divided into two groups, group 1 (*n* 148), their age < 35 years, and group 2 (*n* 70), their age ≥ 35 years. All women received antagonist protocol during their ICSI cycles. Basal FSH and AMH were measured and correlated to the number of follicles on the day of trigger, the number of oocytes retrieved, chemical, and clinical pregnancies.

**Results:**

The fertilization rate in group 1 was 68.15%, while in group 2 was 77.82% (*p* = 0.003) while the implantation rate (number of gestational sacs observed at 6 weeks of pregnancy divided by the number of transferred embryos) was 18.95 and 11.98% in group 1 and group 2, respectively (*p* = 0.041). The clinical pregnancy rate among both groups was 38.51% in group 1, while 24.29% in group 2 (*p* = 0.038). Women who got pregnant among those aged < 35 years had significantly lower basal FSH (*p* < 0.001), while women who got pregnant among those aged ≥ 35 years had significantly higher AMH levels (*p* value < 0.001) and higher E2 levels on the day of trigger (*p* = 0.007).

**Conclusion:**

We found that below the age of 35 years, the chances of pregnancy are more correlated to FSH levels, while above the age of 35 years, AMH was a more relevant test.

## Background

Women’s fecundity is known to decrease with the increase in chronologic age. The age-related decline in women’s fertility is most likely due to the decline in oocytes’ quantity and quality. As a result, researchers started to look for a predictive criterion for fertility [1]. Maternal age provides the best indicator of ovarian reserve. Ovarian reserve and oocyte competence decline when age advances; however, their reliability is unsatisfactory [2]. Personalization of treatment in ICSI should be based on the prediction of ovarian response for every individual. The starting point is to identify if a woman is likely to have a normal, poor, or hyper response and choose the ideal treatment protocol tailored to this prediction [3].

Ovarian reserve refers to the number of primordial follicles in the ovaries that have the ability to develop into mature oocytes [4]. Several biomarkers of the ovarian reserve have been proposed as possible predictors for the response to controlled ovarian stimulation (COS). These markers include ultrasound markers as antral follicle count and ovarian blood supply pulsatility, and resistance index; endocrinal markers as follicle-stimulating hormone (FSH), anti-Müllerian hormone (AMH), and inhibin B; genetic markers as FSH receptor polymorphism, and FMR1 mutation; dynamic tests as the clomiphene citrate challenge test (CCCT), gonadotropin-releasing hormone (GnRH) agonist stimulation test, and exogenous FSH ovarian reserve test [5]. Dynamic tests are more invasive and less convenient for the patient with potential side effects from the administered medications and have now been largely superseded by the more accurate tests, including endocrinal biomarker and AFC. The rest of these biomarkers are now historical to some extent [6, 7]. Baseline FSH level is still one of the commonly used tests for daily practices. It is generally accepted that the ovarian reserve is low when the FSH level exceeds 10–12 IU/L [8].

FSH is a pituitary hormone that acts on women’s ovaries by stimulating granulosa cell proliferation, oocyte maturation, and estrogen synthesis. It is the most widely performed test in women undergoing fertility treatment [9]. AMH is a dimeric glycoprotein member of the TGF-β family. In women, AMH is derived primarily from preantral and early antral follicles and has been shown in recent years to accurately reflect the follicular pool [10]. Although the AMH level is used to assess the ovarian reserve in many scenarios, it has served most commonly to assess the likelihood of an adequate response during ovarian stimulation for assisted reproduction. AMH is predictive of the number of oocytes retrieved and increasingly is being used to guide the selection of the stimulation protocol [11].

Although the considerable focus has been on the performance of biomarkers, in reality, the success of assisted conception is modified by a variety of baseline characteristics of each individual. These in themselves may be indirectly associated with ovarian reserve; for example, advancing maternal age, previous unsuccessful cycles, increasing duration of infertility, and tubal or anovulatory cause of infertility all independently decreased the odds of a successful live birth per cycle [12].

Although there are assumptions indicating that the relationship between age and ovarian reserve is highly variable and the different potential validity of ovarian reserve markers in women in different age groups remains to be demonstrated. There are not enough studies assessing the predictive value of these markers in different age strata since some ovarian reserve markers may have different accuracy in different ages [13].

## Methods

This prospective study has been carried out on women having ICSI cycles over a period of 24 months, from April 2019 till March 2021. The initially proposed time frame for the study was to take only 12 months, but due to the COVID-19 pandemic and the marked decrease in cases having ICSI, we extended the study time to collect the planned number of cases according to the study design. Ethical approval has been taken before starting the study. The Ethical Committee of the National Research Centre approved the study.

Written consents had been taken from all women who were willing to participate in the study. They were recruited from the Medical Research Centre of Excellence (National Research Centre), Kasr El Eini, and private infertility outpatient clinics. All women included in the study had a BMI 20–30 kg/m^2^, age 20–44 years, 1ry or 2ry infertility, non-smokers for the past 6 months, no hormonal treatment in the last 3 months preceding the ICSI trial.

Women with any of the following criteria have been excluded from the study; polycystic ovarian syndrome (PCOS), endometriosis, known pelvic pathology as uterine fibroids or ovarian masses, uterine anomalies as uterine septum, smokers within the last 6 months, known autoimmune disease, diabetics, previous ovarian surgery that may have affected ovarian functions, history of previous chemo or radiotherapy, history of COVID-19 infection (unknown effect on ovarian functions), and recurrent implantation failure (cases of previous 3 ICSI failures despite transferring good quality embryos).

All women had a basal FSH/AMH/E2 measured between days 2 and 4 of the menstrual cycle in the month preceding their ICSI cycles. Serum FSH/AMH/E2 were measured by enzyme-linked immunosorbent assay (ELISA) kit (Immuno-tech-Beckman Coulter, Webster, TX, USA) and expressed in ng/ml, IU/ml, and pg/ml, respectively. Also, antral follicle count (AFC) has been measured using transvaginal ultrasound.

Cases were divided into two groups, group A (*n* = 148) with age < 35 years, and group B (*n* = 70) with age ≥ 35 years. According to our protocol, all women received antagonist protocol during their ICSI cycles to decrease the possibility of developing ovarian hyperstimulation syndrome. Controlled ovarian stimulation has been done using highly purified human urofollitropin intramuscular or subcutaneous injections with doses between 150 and 450 IU/day (Fostimon, IBSA, Switzerland) starting on day 2 the cycle and the dose adjusted according to weight, age, and ovarian response. In our practice, we adopt the antagonist fixed protocol, so cetrorelix 0.25 mg/day subcutaneous (*Cetrotide, Merck Serono, Germany*) was started on day 6 of ovarian stimulation. Women were followed using transvaginal ultrasound and serial E2 measures. The number of mature follicles > 15 mm and the level of E2 on the day of the trigger were documented.

Human chorionic gonadotropin (HCG) 10,000 IU trigger shot was given intramuscularly when at least 2 dominant follicles have reached 18–20 mm. Ovum pickup under transvaginal ultrasound guidance was done 34–36 h following the trigger shot. Women with a high level of E2 and a large number of follicles impending to develop ovarian hyperstimulation syndrome (OHSS) was given gonadotropin-releasing hormone agonist 0.2 mg triptorelin acetate subcutaneous injection (Decapeptyl, Ferring, Switzerland) as a trigger shot and excluded from the final analysis. The cycle was cancelled when there were < 3 follicles with diameter < 14 mm after 8–9 days of gonadotropin therapy or after 4–5 additional treatment days without attaining the criteria for HCG administration.

After 12–24 h from oocytes retrieval, oocytes were checked for fertilization. Embryos were transferred on day 3. According to the number and quality of embryos and under abdominal ultrasound guidance, all patients had a maximum of four embryos transferred per cycle. The maturational status of the oocytes and the embryo grading was recorded according to published criteria [14]; embryos of Veeck grades 1 or 2 were considered high quality and thus suitable for transfer. Embryo transfer was done without anesthesia or sedation using a soft catheter. Luteal support was given using intramuscular progesterone 100 mg once daily starting from the day of ovum pickup.

Women were instructed to have a blood pregnancy test 14 days after embryo transfer, and if positive, they had an ultrasound 2 weeks later to check viability and number of fetuses.

### Statistical analysis

Statistical analyses were performed using the SPSS software (SPSS, version 25, SPSS, Inc., IL, USA). The distribution of the measured variables was determined using the Shapiro test. Normally distributed variables were presented as means ± standard deviation (SD), while non-normally distributed variables were presented as medians and interquartile ranges (IQR). Statistical significance of differences for normally distributed variables was tested using the Student’s *t* test, while the statistical significance of differences for non-normally distributed variables was tested using the Mann–Whitney test. Categorical data differences were compared by the chi-square test. Correlation analysis between the numerical variables was done using Pearson correlation. For all statistical tests, *p* values were considered statistically significant if less than 0.05.

### Outcome measures

Our primary outcomes were the number of follicles on the day of trigger and the number of oocytes retrieved, while the secondary outcomes were the chemical and clinical pregnancies.

## Results

Among 234 women who had ICSI cycles using antagonist protocol, 218 women went for embryo transfer and completed the study. The embryo transfer step was cancelled, and the decision to freeze all embryos was taken in 10 cases due to either pending OHSS (7 patients), thin endometrium < 6 mm (3 patients), or cycle cancelled in 6 poor responders. The average age of the participants (218 women) was 31.24 ± 6.1 years, BMI was 26.5 ± 3.1 kg/m^2^, and the duration of infertility was 5 ± 3.7 years. The average numbers of follicles detected by ultrasound on the day of trigger, retrieved oocytes, and injected oocytes were 8.9, 7.2, and 5.3, respectively. In group 1, the average number of follicles on the day of trigger, retrieved oocyte, and injected oocytes was 10, 8, and 5, while in group 2, they were 5, 4, and 3, respectively. Embryo transfer was done on day 3. The average number of frozen embryos among women who had a chance to freeze embryos (*n* = 59) was 3.54.

The corrected fertilization rate, “the number of fertilized oocytes divided by the total number of metaphase II oocytes,” was 70.3% (group 1 68.15 vs group 2 77.82% and *p* = 0.003), while the implantation rate, “the number of gestational sacs observed at 6 weeks of pregnancy divided by the number of transferred embryos” was 17% (group 1 18.95 vs group 2 11.98% and *p* = 0.041). Out of the 218 participating women, 74 women got pregnant, accounting for a clinical pregnancy rate of 34%. Looking closely at the clinical pregnancy rate among both groups, it was 38.51% in group 1 while 24.29% in group 2 (*p* = 0.038) (Table 1).

**Table 1:** Comparison between the two age groups; group 1 included women < 35 years, and group 2 included women ≥ 35 years

	**“Group 1”** **(*n* = 148)**	**“Group 2”** **(*n* = 70)**	***P* value**
Age (years)	27.82 ± 3.98	38.47 ± 2.06	< 0.001*
BMI (kg/m^2^)	26.57 ± 3.08	26.48 ± 3.17	0.846
Duration of infertility (years)	4 (3,6)	4 (3,7)	0.348
Antral follicular count	12 (8,18)	7 (5,9.75)	< 0.001*
Basal FSH (IU/L)	5.8 (4.8,7.6)	7.125 (5.3,9.375)	< 0.001*
Basal E_2_ (pg/ml)	42.5 (31,57.5)	47.5 (37,68.75)	0.062
AMH (ng/ml)	2.55 (1.515,3.8025)	1.1 (0.6,1.9)	< 0.001*
E_2_ on day of trigger (pg/ml)	2509 (1603.75,3961.5)	1500 (964,2091.25)	< 0.001*
Days of induction	12 (10.25,13)	11 (10,12.75)	0.067
No. of induction ampoules	39 (33,51)	50 (40,60)	< 0.001*
No. of obtained follicles	10 (6,14)	5 (3,7.75)	< 0.001*
No. of oocytes retrieved	8 (5,10)	4 (2,6)	< 0.001*
No. of oocytes injected	5 (4,8)	3 (2,5)	< 0.001*
No. of transferred embryos	3 (2,4)	2 (2,3)	< 0.001*
No. of frozen embryos	0 (0,2)	0 (0,0)	0.013*
Fertilization rate	614/901 (68.15%)	200/257 (77.82%)	0.003*
Implantation rate	83/438 (18.95%)	20/167 (11.98%)	0.041*
Clinical pregnancy rate	57 (38.51%)	17 (24.29%)	0.038*

^*^p value is significant

Compared to women in “group 2”, women in “group 1”, had a significantly higher number of AFC, lower level of basal FSH, higher level of AMH, and higher E_2_ level on the day of trigger. Although the number of ampoules used in ovulation induction for women in group 2 was more than in group 1, the number of obtained follicles in group 2 was fewer than in group 1, with a consequently fewer number of the oocytes retrieved and injected.

Correlation analysis between the basal hormone levels (FSH and AMH) and the number of obtained follicles after induction was done in each group. Women < 35 years showed a significant –ve correlation between the basal FSH level and the number of follicles (*r* =  − 37, *p* < 0.01) and a significant + ve correlation between the AMH level and the number of follicles (*r* = 37, *p* < 0.01). Also, women ≥ 35 years showed the same significant –ve correlation between the basal FSH level and the number of follicles (*r* =  − 46, *p* < 0.01) and a significant + ve correlation between the AMH level and the number of follicles (*r* = 60, *p* < 0.01).

**Fig. 1:**
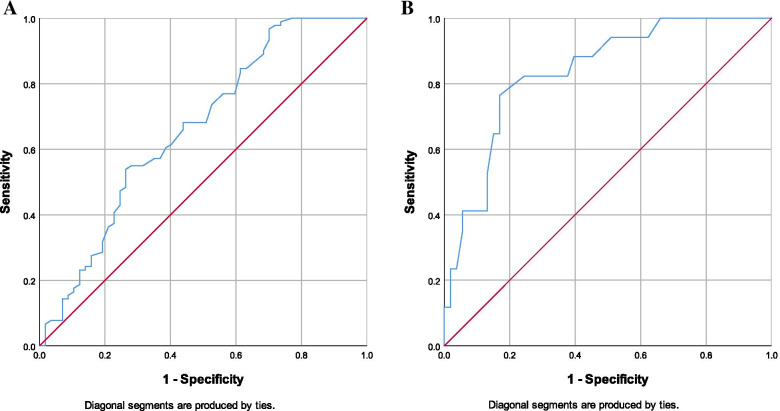
**A** ROC curve of FSH for women < 35 years.** B** ROC curve of AMH for women ≥ 35 years

Thus, to reach out the hormone that most influences the pregnancy rate in each age group, we subdivided each group according to their outcome; if clinical pregnancy occurred or not (Tables 2 and 3). Interestingly, women who got pregnant among those aged < 35 years had significantly lower basal FSH levels (5.3 IU/ml in pregnant women vs 6.3 in non-pregnant women and *p* value < 0.001), while women who got pregnant among those aged ≥ 35 years had significantly higher AMH levels (2.1 ng/ml in pregnant women vs 0.8 in non-pregnant women and *p* value < 0.001) and higher E_2_ levels on the day of trigger (1987 pg/ml in pregnant women vs 1378 in non-pregnant women and *p* value = 0.007). Therefore, ROC curves were performed for FSH in group 1 and AMH in group 2 (Fig. 1).

**Table 2:** Comparison between women who got pregnant and those who did not get (among women < 35 years, group 1)

	**Women who got pregnant among women < 35 years** **(*n* = 57)**	**Women who did not get pregnant among women < 35 years (*n* = 91)**	***P* value**
Age (years)	27.84 ± 4.22	27.81 ± 3.84	0.966
BMI (kg/m^2^)	26.68 ± 3.36	26.50 ± 2.91	0.740
Duration of infertility (years)	3 (2.5,6)	4 (3,6.75)	0.128
Antral follicular count	13.5 (10,18)	12 (8,18)	0.188
Basal FSH (IU/L)	5.3 (3.7,6.325)	6.3 (5,8.3)	< 0.001*
Basal E_2_ (pg/ml)	43.5 (29.25,52.15)	42 (31,59)	0.950
AMH (ng/ml)	2.55 (1.78,3.9675)	2.55 (1.3,3.6)	0.488
E_2_ on day of trigger (pg/ml)	2675.5 (1751,4302.5)	2344 (1558,3586.75)	0.083
Days of induction	12 (11,13)	12 (10,13)	0.963
No. of induction ampoules	39 (33,50.25)	38 (33,51.75)	0.847
No. of obtained follicles	12 (8,14.75)	8 (6,13.75)	0.007*
No. of oocytes retrieved	10 (6,10.75)	7 (4.25,10)	0.020*
No. of oocytes injected	6 (5,9)	5 (3,6.75)	< 0.001*
No. of transferred embryos	3 (2,4)	3 (2,3)	0.057
No. of frozen embryos	0 (0,2)	0 (0,2)	0.180
Fertilization rate	263/395 (66.58%)	351/506 (69.37%)	0.373

^*^p value is significant

**Table 3:** Comparison between women who got pregnant and those who did not get (among women ≥ 35 years, group 2)

	**Women who got pregnant among women ≥ 35 years** **(*n* = 17)**	**Women who did not get pregnant among women ≥ 35 years (*n* = 53)**	***P* value**
Age (years)	38.18 ± 2.32	38.57 ± 1.98	0.501
BMI (kg/m^2^)	26.80 ± 2.87	26.38 ± 3.28	0.637
Duration of infertility (years)	4 (2.5,6)	4 (3,9)	0.568
Antral follicular count	8 (6,11.5)	6 (5,8)	0.037*
Basal FSH (IU/L)	6.1 (5.1,7.55)	7.9 (5.3,9.9)	0.074
Basal E_2_ (pg/ml)	50 (38.5,76.5)	46 (32,68)	0.376
AMH (ng/ml)	2.1 (1.65,2.95)	0.8 (0.5,1.53)	< 0.001*
E_2_ on day of trigger (pg/ml)	1987 (1591,2522.5)	1378 (945,1965)	0.007*
Days of induction	11 (10,12.5)	11 (10,13)	0.939
No. of induction ampoules	45 (36,57.5)	52 (40,65)	0.391
No. of obtained follicles	8 (5.5,9.5)	4 (3,7)	< 0.001*
No. of oocytes retrieved	6 (4.5,8)	3 (2,5)	< 0.001*
No. of oocytes injected	6 (3,7)	3 (2,4)	< 0.001*
No. of transferred embryos	3 (3,3)	2 (1,3)	0.007*
No. of frozen embryos	0 (0,2.5)	0 (0,0)	0.003*
Fertilization rate	69/92 (75.00%)	131/165 (79.39%)	0.416

^*^p value is significant

## Discussion

Nowadays, there is an increasing number of babies born as a result of assisted reproductive techniques (ART). When contemplating an ICSI cycle, women would like to inquire about their chances of getting pregnant. Several markers have been used to predict the outcome before embarking on an ICSI cycle, with AMH and FSH are the most popular. AFC is widely used as a predictor for ovarian response [15], but there are inter-observer variations based on observer experience. Applicability of FSH and AMH were independently demonstrated in different studies to evaluate IVF success, while others studies evaluated the discordant and concordant combinations of AMH and FSH measurements to predict ovarian responses in women undergoing ART [16]. In our practice, we faced many women who had marked discrepancy between FSH and AMH levels. In this study, we tried to solve this dilemma by studying basal FSH and AMH levels in women having ICSI cycles according to their age to find which is more relevant and predictive of ICSI results, FSH or AMH.

Our primary outcomes were the number of follicles on the day of trigger and the number of oocytes retrieved, while the secondary outcomes were the chemical and clinical pregnancies.

When comparing the two groups according to their age, there was a significant difference between the two groups in terms of pregnancy rate (chemical and clinical), number of follicles, and number of oocytes retrieved and injected. Nevertheless, the fertilization rate was lower in the younger group.

Antagonist protocol can cause asynchronous growth of follicles with an early leading dominant follicle and heterogeneous size follicle cohort due to the lack of endogenous down-regulation like in long agonist protocol [17]. Marked discrepancies in the size of follicles could be related to differences in follicles sensitivity to FSH and un-satisfactory maturation. This phenomenon potentially causes a decrease in the number of viable oocytes and embryos [18]. In young women, the number of follicles in response to ovarian stimulation was larger; however, a percentage of them was small follicles giving immature oocytes. This affected the fertilization rate in this group, despite having a higher pregnancy rate. Also, Abdallah’s research team [19] found that the fertilization rate was not affected by the level of basal FSH or the age of the women.

In both groups, women who got pregnant and those who did not get pregnant, there was a significant difference between the number of follicles seen on the day of trigger, the number of oocytes retrieved, and the number of oocytes injected, while the fertilization rate did not significantly differ.

After analyzing our data, we found out that in women below 35 years, basal FSH level was more correlated to the number of follicles and number of oocytes retrieved, which in turn reflected on pregnancy rate. On the other hand, AMH, rather than FSH, was more correlated to the same outcomes in women aged ≥ 35 years.

FSH reflects the number of follicles, while age is a qualitative measure of ovarian reserve [20]. According to our results, we expect that women under 35 would have good quality oocytes in most cases, and FSH predicts the number of follicles, which can be translated to pregnancy rate.

Moez Kudos et al. [21], in their study on 676 women undergoing ICSI, found that high basal FSH levels in patients < 38 years could predict a more poor response, an increased number of cancelled cycles and a lower number of oocytes retrieved, which in turn resulted in lower embryos obtained but this finding did not cause to reflect a lower pregnancy, childbirth, or implantation rate. In their study on 361 women with AMH level < 0.5 ng/ml, Revelli and his colleagues found young patients with very low AMH levels still have reasonable chances of successful pregnancy with IVF [22]. This matches with our findings that AMH is not a sensitive predictor in younger women.

Our results agreed with Gomez et al. (2016), who stated that in women above 36 years, AMH could predict pregnancies; however, it is not an important predictor in younger women [22]. The positive correlation between IVF/ICSI success and ovarian reserve measured by serum AMH could have variable importance according to the patient’s age [23]. Also, Hanan El Anazi and her coworkers studied 258 women who had a poor ovarian response and found that AMH predicted a lower number of pregnancies in the cohort of women studied. In women above 35 years, we expect a considerable percentage of those women to be poor responders [24].

AMH level was previously suggested as a predictor of menopause, and very low, even undetectable, levels could be seen up to the 5 years preceding the menopause. As AMH levels decline earlier than FSH, the predictive value of FSH levels as a determinant of aging predictor is lower than that of AMH [25, 26]. Ligon et al. (2019) evaluated discordant and concordant values of AMH and FSH on live birth rate and IVF cycle cancellation rate. The live birth rate of patients with normal AMH and elevated FSH was higher than those of patients with low AMH and normal FSH (39 vs. 26%). The live birth rate in patients with normal AMH and normal FSH (concordant) was higher than in any other group (44%). Besides, the IVF cycle cancellation rate in patients with normal AMH and FSH was lower than that of other groups (4%), and this rate was higher in patients with elevated FSH and low AMH compared to other groups (30%) [27].

Also, the relationship between basal FSH and AMH with IVF/ICSI success changes with maternal age; basal FSH better reflects clinical outcomes probably determined by oocyte quality in women < 35 years, while AMH better suits patients ≥ 35 years[28].

On the contrary, both Kedem and his team [29], as well as Lukaszuk’s study group [30], had found that even with extremely low AMH, pregnancy is possible, and AMH should not be used as a predictor for the decision of ICSI. However, in those studies, they compared women with low and extremely low AMH, which could explain the difference between their results and ours.

The ultimate goal of ART is to get a live birth, not just getting pregnant, so one of the limitations of this study is that we were not able to follow-up women till they give birth to observe any antenatal complications. The reason was that many of our cases came from remote areas to have ICSI cycles then left back to continue their antenatal care in places close to their residencies.

The vast majority of research done on this topic studied FSH/AMH in poor responders or linked those markers to the outcome without correlating those markers to age as we did in our study.

## Conclusions

FSH and AMH are widely used as predictive markers to forecast the chances of pregnancy before ICSI cycles. We found that below the age of 35 years, the chances of pregnancy are more correlated to FSH levels, while above the age of 35 years, AMH was more reliable. Nevertheless, the decision to discourage women from having ICSI cycles depending solely on FSH and AMH cannot be justified. Further, large prospective studies are needed to expound and confirm these findings and prior to its routine implementation.

## Abbreviations

AFC: Antral follicle count
AMH: Anti-Mullerian hormone
ART: Assissted reproductive techniques
BMI: Body mass index
CCCT: Clomiphene citrate challenge test
COS: Controlled ovarian stimulation
E2: Estradiol
ELISA: Enzyme-linked immunosorbent assay
FSH: Follicle-stimulating hormone
GnRH: Gonadotropin-releasing hormone
HCG: Human chorionic gonadotropin
ICSI: Intra-cytoplasmic sperm injection
IVF: In vitro fertilization
OHSS: Ovarian hyperstimulation syndrome
PCOS: Polycystic ovarian syndrome

## References

[1] Mutlu MF, Erdem M, Erdem A (2013). Antral follicle count determines poor ovarian response better than anti-Müllerian hormone but age is the only predictor for live birth in in vitro fertilization cycles. J Assist Reprod Genet.

[2] Medicine P.C. of the AS for R (2015). Testing and interpreting measures of ovarian reserve: a committee opinion. Fertil Steril.

[3] La Marca A, Sunkara SK (2014). Individualization of controlled ovarian stimulation in IVF using ovarian reserve markers: from theory to practice. Hum Reprod Update.

[4] Qiao J, Wang Z-B, Feng H-L (2014). The root of reduced fertility in aged women and possible therapentic options: current status and future perspects. Mol Aspects Med.

[5] Iliodromiti S, Nelson SM (2013). Biomarkers of ovarian reserve. Biomark Med.

[6] La Marca A, Argento C, Sighinolfi G (2012). Possibilities and limits of ovarian reserve testing in ART. Curr Pharm Biotechnol.

[7] Tal R, Seifer DB (2017). Ovarian reserve testing: a user’s guide. Am J Obstet Gynecol.

[8] Fang T, Su Z, Wang L (2015). Predictive value of age-specific FSH levels for IVF-ET outcome in women with normal ovarian function. Reprod Biol Endocrinol.

[9] Tsepelidis S, Devreker F, Demeestere I (2007). Stable serum levels of anti-Müllerian hormone during the menstrual cycle: a prospective study in normo-ovulatory women. Hum Reprod.

[10] Broer SL, Broekmans FJ, Laven JS, Fauser BC (2014). Anti-Müllerian hormone: ovarian reserve testing and its potential clinical implications. Hum Reprod Update.

[11] Amer SA, Mahran A, Abdelmaged A (2013). The influence of circulating anti-Müllerian hormone on ovarian responsiveness to ovulation induction with gonadotrophins in women with polycystic ovarian syndrome: a pilot study. Reprod Biol Endocrinol.

[12] Nelson SM, Lawlor DA (2011). Predicting live birth, preterm delivery, and low birth weight in infants born from in vitro fertilisation: a prospective study of 144,018 treatment cycles. PLoS Med.

[13] Tehraninezhad ES, Mehrabi F, Taati R (2016). Analysis of ovarian reserve markers (AMH, FSH, AFC) in different age strata in IVF/ICSI patients. Int J Reprod BioMed.

[14] Veeck LL (1999). An atlas of human gametes and conceptuses: an illustrated reference for assisted reproductive technology.

[15] Broekmans FJ, Verweij PJ, Eijkemans MJ (2014). Prognostic models for high and low ovarian responses in controlled ovarian stimulation using a GnRH antagonist protocol. Hum Reprod.

[16] Güngör ND, Gürbüz T (2020). Prediction of the number of oocytes based on AMH and FSH levels in IVF candidates. J Surg Med.

[17] Depalo R, Jayakrishan K, Garruti G (2012). GnRH agonist versus GnRH antagonist in in vitro fertilization and embryo transfer (IVF/ET). Reprod Biol Endocrinol.

[18] Opsahl MS, Blauer KL, Black SH (2001). The number of embryos available for transfer predicts successful pregnancy outcome in women over 39 years with normal ovarian hormonal reserve testing. J Assist Reprod Genet.

[19] Abdalla H, Thum MY (2004). An elevated basal FSH reflects a quantitative rather than qualitative decline of the ovarian reserve. Hum Reprod.

[20] Dua M, Bhatia V, Malik S, Prakash V (2013). ART outcome in young women with premature ovarian aging. J Mid-Life Health.

[21] Kdous M, Merdassi G, Zhioua F (2016). Basal follicle stimulating hormone level correlated to age is a good prognostic criterion for the outcome of intracytoplasmic sperm microinjection. Tunis Med.

[22] Gomez R, Schorsch M, Hahn T (2016). The influence of AMH on IVF success. Arch Gynecol Obstet.

[23] Revelli A, Biasoni V, Gennarelli G (2016). IVF results in patients with very low serum AMH are significantly affected by chronological age. J Assist Reprod Genet.

[24] Alanazi H, Bushaqer N, Ayyoub H (2018). Antimullerian hormone (AMH) level and IVF/ICSI cycle outcome in expected poor responders. Middle East Fertil Soc J.

[25] Depmann M, Eijkemans MJC, Broer SL (2016). Does anti-Müllerian hormone predict menopause in the general population? Results of a prospective ongoing cohort study. Hum Reprod.

[26] Kruszyńska A, S\lowińska-Srzednicka J (2017). Anti-Müllerian hormone (AMH) as a good predictor of time of menopause. Menopause Rev.

[27] Ligon S, Lustik M, Levy G, Pier B (2019). Low antimüllerian hormone (AMH) is associated with decreased live birth after in vitro fertilization when follicle-stimulating hormone and AMH are discordant. Fertil Steril.

[28] Qiao J, Wang ZB, Feng HL, Miao YL, Wang Q, Yu Y, Wei YC, Yan J, Wang WH, Shen W (2014). The root of reduced fertility in aged women and possible therapentic options: current status and future perspects. Mol Asp Med.

[29] Kedem A, Haas J, Geva LL (2013). Ongoing pregnancy rates in women with low and extremely low AMH levels. A multivariate analysis of 769 cycles. PLoS One.

[30] Lukaszuk K, Kunicki M, Liss J (2014). Probability of live birth in women with extremely low anti-Müllerian hormone concentrations. Reprod Biomed Online.

